# *catena*-Poly[[[diaqua­cadmium(II)]-bis­[μ-3,5-bis­(isonicotinamido)benzoato]] tetra­hydrate]

**DOI:** 10.1107/S1600536810036147

**Published:** 2010-09-11

**Authors:** Man-Sheng Chen, Yi-Fang Deng, Chun-Hua Zhang, Dai-Zhi Kuang

**Affiliations:** a Key Laboratory of Functional Organometallic Materials, Department of Chemistry and Materials Science, Hengyang Normal University, Hengyang, Hunan 421008, People’s Republic of China

## Abstract

The title compound, {[Cd(C_19_H_13_N_4_O_4_)_2_(H_2_O)_2_]·4H_2_O}_*n*_ or {[Cd(BBA)_2_(H_2_O)_2_]·4H_2_O}_*n*_, where BBA is 3,5-bis­(iso­nicotin­amido)­benzoate, is isotypic with its Mn isologue [Chen *et al.* (2009 ▶). *J. Coord. Chem.* **62**, 2421–2428]. The cation sits on a twofold axis and is six-coordinated in a slightly distorted octa­hedral geometry; the polyhedra are linked into zigzag chains, which are further connected by N—H⋯O, O—H⋯O and O—H⋯N hydrogen bonds as well as π–π inter­actions [centroid-centroid distance of 3.639 (2) Å], giving a three-dimensional supra­molecular framework.

## Related literature

For the isotypic Mn structure, see: Chen *et al.* (2009 ▶). For the properties of coordination polymers, see: Evans & Lin (2002 ▶); Yaghi *et al.* (2003 ▶); Kitagawa *et al.* (2004 ▶); Biradha *et al.* (2006 ▶); Wu *et al.* (2009 ▶). For the rational design and synthesis of new supra­molecular frameworks by covalent and weak intra/inter­molecular inter­actions, see: Eddaoudi *et al.* (2001 ▶); Moulton & Zaworotko (2001 ▶); Cheng *et al.* (2002 ▶); Zhang *et al.* (2003 ▶); Go *et al.* (2004 ▶). For the coordination capacities of carboxyl­ate, pyridine and amide groups, see: Bent (1968 ▶); Huyskens (1977 ▶); Lee & Kumler (1962 ▶); Wang *et al.* (2007 ▶).
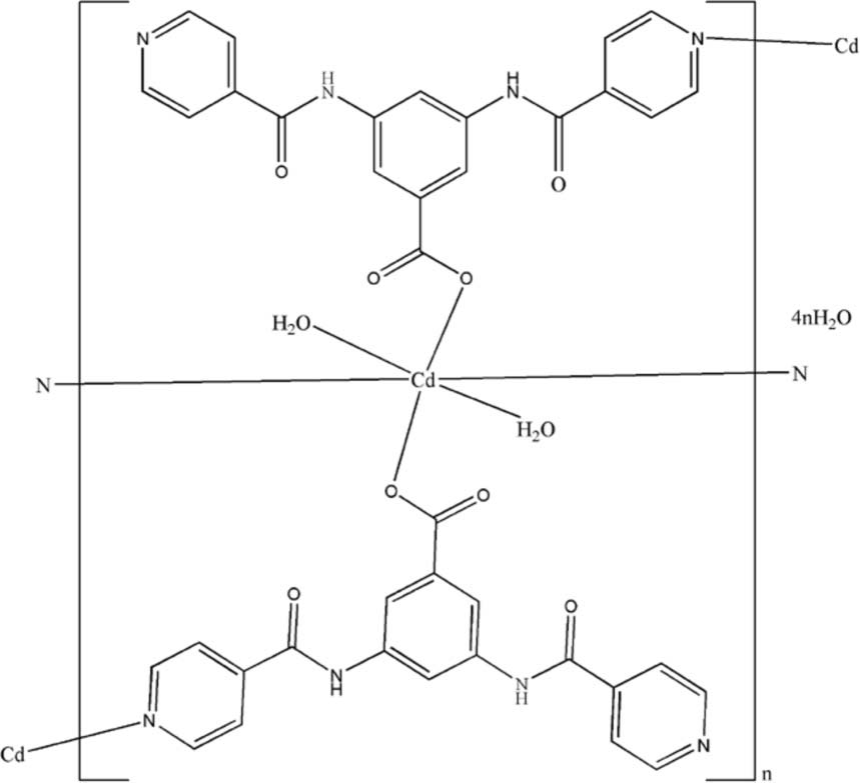


## Experimental

### 

#### Crystal data

[Cd(C_19_H_13_N_4_O_4_)_2_(H_2_O)_2_]·4H_2_O
*M* *_r_* = 943.16
Monoclinic, 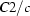

*a* = 17.584 (3) Å
*b* = 10.8568 (19) Å
*c* = 21.891 (4) Å
β = 103.801 (2)°
*V* = 4058.5 (12) Å^3^
*Z* = 4
Mo *K*α radiation
μ = 0.62 mm^−1^
*T* = 293 K
0.20 × 0.16 × 0.10 mm

#### Data collection

Bruker SMART APEX CCD diffractometer
Absorption correction: multi-scan (*SADABS*; Bruker, 2000 ▶) *T*_min_ = 0.887, *T*_max_ = 0.941
10338 measured reflections
3867 independent reflections
3391 reflections with *I* > 2σ(*I*)
*R*_int_ = 0.055

#### Refinement

*R*[*F* ^2^ > 2σ(*F* ^2^)] = 0.047
*wR*(*F* ^2^) = 0.110
*S* = 1.08
3867 reflections
300 parameters
13 restraints
H atoms treated by a mixture of independent and constrained refinement
Δρ_max_ = 0.80 e Å^−3^
Δρ_min_ = −0.48 e Å^−3^

### 

Data collection: *SMART* (Bruker, 2000 ▶); cell refinement: *SAINT* (Bruker, 2000 ▶); data reduction: *SAINT*; program(s) used to solve structure: *SHELXTL* (Sheldrick, 2008 ▶); program(s) used to refine structure: *SHELXTL*; molecular graphics: *SHELXTL*; software used to prepare material for publication: *SHELXTL*.

## Supplementary Material

Crystal structure: contains datablocks global, I. DOI: 10.1107/S1600536810036147/bg2362sup1.cif

Structure factors: contains datablocks I. DOI: 10.1107/S1600536810036147/bg2362Isup2.hkl

Additional supplementary materials:  crystallographic information; 3D view; checkCIF report

## Figures and Tables

**Table 1:** Hydrogen-bond geometry (Å, °)

*D*—H⋯*A*	*D*—H	H⋯*A*	*D*⋯*A*	*D*—H⋯*A*
N3—H3⋯O6	0.86	2.10	2.885 (5)	151
N4—H4⋯O3^i^	0.86	2.26	3.096 (4)	164
O5—H5*B*⋯O2	0.83 (3)	1.98 (3)	2.710 (4)	145 (4)
O5—H5*A*⋯N1^ii^	0.83 (2)	2.00 (2)	2.825 (4)	172 (4)
O6—H6*B*⋯O5^iii^	0.84 (2)	2.40 (4)	3.163 (6)	151 (6)
O6—H6*A*⋯O7	0.82 (3)	1.92 (4)	2.681 (6)	148 (5)
O7—H7*A*⋯O2^iv^	0.85 (2)	1.96 (3)	2.767 (4)	162 (5)
O7—H7*B*⋯O4^v^	0.84 (2)	1.99 (3)	2.791 (4)	159 (6)

Symmetry codes: (i) 
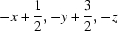
; (ii) 
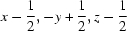
; (iii) 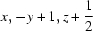; (iv) 
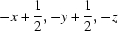
; (v) 
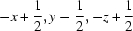
.

**Fig. 1.**
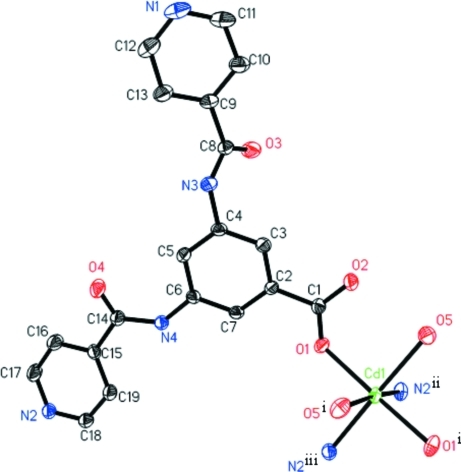
The ORTEP drawing of the title compound (I). Displacement ellipsoids are drawn at 30% probability level [symmetry code: (i) -x, y,-z - 1/2 (ii) x, 2 - y, -1/2 + z (iii) -x, 2 - y,-z].

**Fig. 2.**
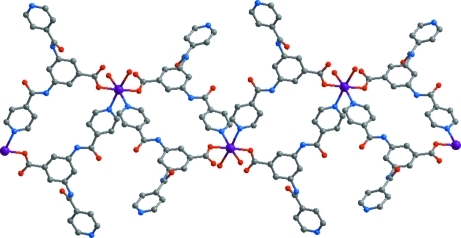
Projection showing the one-dimensional structure of the compound (I).

## supplementary crystallographic information

### Comment

Coordination polymeric structures are one of the most attractive areas of materials research due to their intriguing structural topologies and functional properties such as molecular adsorption, magnetism and luminescence (Evans & Lin, 2002; Yaghi *et al.*, 2003; Kitagawa *et al.*,2004; Biradha *et al.*, 2006; Wu *et al.*, 2009) but in spite of this interest the rational design and synthesis of new supramolecular frameworks by covalent and weak intra/intermolecular interactions is still a challenge (Eddaoudi, *et al.* 2001; Moulton & Zaworotko, 2001; Cheng, *et al.* 2002; Zhang, *et al.* 2003; Go, *et al.* 2004). On the other hand, it is well known that carboxylate and pyridine groups have good coordination capacities as well as the amide group, a fascinating functional group with two different types of hydrogen bonding sites: the –NH moiety which acts as an electron acceptor while the –C=O group acts as an electron donor (Lee, *et al.*, 1962; Bent, 1968; Huyskens, 1977; Wang, *et al.*, 2007). we have recently pursued systematic investigations into the assembly of polymers through ligands containing both carboxylate and amido-pyridine groups, in order to study the influence of different metal ions. In the present work we report the structure of a new cadmium coordination polymer of the bridging ligand 3,5-bis(isonicotinamido)benzoate (BBA^-^), namely {[Cd(BBA)_2_(H_2_O)_2_].4(H_2_O)}_n_, (I), which is isomorphous to its Mn isologue [Mn(BBA)_2_(H_2_O)_2_](Chen, *et al.* 2009).

As shown in Fig. 1, the asymmetric unit of (I) is composed of one-half of a Cd^II^ cation, which sits on a crystallographic twofold axis, one BBA^-^ ligand, one coordinated water molecule and two solvent water molecules. The central Cd^II^ atom has the [CdN_2_O_4_] octahedral coordination geometry with four coordination sites in one plane occupied by two *cis*-positioned N atoms from two BBA^-^ ligands ligands and two water molecules, and the other two coordination sites taken up by two *trans*-positioned carboxylate O atoms from two further BBA^-^ ligands. The ligand (3,5-bis(isonicotinamido)benzoic acid) is fully deprotonated(BBA^-^) and the carboxylate group adopts a monodentate mode, coordinating only through one O atom to one Cd^II^ centre. On the other hand, the dihedral angles between the central benzene and terminal pyridine ring are 15.66 (10) ° and 19.72 (11)°, respectively.

It is noteworthy that each BBA^-^ ligand in turn uses its carboxylate group and one of the two pyridinyl groups to connect two metal centers, while the other pyridinyl group does not coordinate. Then, two Cd(II) and two BBA^-^ ligands form a Cd_2_(BBA)_2_ macrocyclic ring with Cd···Cd distance of 12.313 (18) Å. Such *M*_2_(BBA)_2_ macrocyclic rings are further connected by Cd—N and Cd—O coordination bonds to give an infinite one-dimensional zigzag chain structure(Fig. 2).

In addiiton, there are non-bonding interactions which consolidate the framework structure, in particular some O—H···O, N—H···O and O—H···N hydrogen bonds (Table 1) as well as π-π interactions between the central benzene ring of *BBA*^-^ anions (C2-->C7) and its symmetry related counterpart, symmetry code:1/2 - *x*,3/2 - *y*,-*z*) with a centroid-centroid distance of 3.639 (2) Å, and slippage and interplanar distances of 1.514 and 3.308 Å, respectively.

### Experimental

All reagents and solvents were used as obtained commercially without further purification. A mixture containing Cd(NO_3_)_2_.6H_2_O (31.1 mg, 0.1 mmol), HL (36.5 mg, 0.12 mmol), N(CH_2_CH_3_)_3_ (0.5 mL, 0.1 mmol), 10 ml H_2_O was sealed in a 16 ml Teflon-lined stainless steel container and heated at 393 K for 3 days. After cooling to room temperature within 12 h, block colorless crystals of (I) suitable for X-ray diffraction analysis were obtained in 39% Yield. Anal. Calcd for C_38_H_38_CdN_8_O_14_: C, 48.39; H, 4.06; N, 11.88%; Found: C, 48.43; H, 4.11; N, 11.76%.

### Refinement

H atoms bonded to C atoms were placed geometrically and treated as riding, with C—H distances 0.93 Å and *U*_iso_(H) = 1.2*U*_eq_(C). The H atoms of water molecules were determined from a difference Fourier synthesis and refined with restrained O—H distances 0.85 (2) Å. The amide H atoms were located from difference maps and refined with the N—H distances restrained to 0.8600 Å.

### Crystal data

**Table:**

[Cd(C_19_H_13_N_4_O_4_)_2_(H_2_O)_2_]·4H_2_O	*F*(000) = 1928
*M**_r_* = 943.16	*D*_x_ = 1.544 Mg m^−^^3^
Monoclinic, *C*2/*c*	Mo *K*α radiation, λ = 0.71073 Å
Hall symbol: -C 2yc	Cell parameters from 3272 reflections
*a* = 17.584 (3) Å	θ = 2.3–25.3°
*b* = 10.8568 (19) Å	µ = 0.62 mm^−^^1^
*c* = 21.891 (4) Å	*T* = 293 K
β = 103.801 (2)°	Block, colorless
*V* = 4058.5 (12) Å^3^	0.20 × 0.16 × 0.10 mm
*Z* = 4	

### Data collection

**Table:**

Bruker SMART APEX CCD diffractometer	3867 independent reflections
Radiation source: fine-focus sealed tube	3391 reflections with *I* > 2σ(*I*)
graphite	*R*_int_ = 0.055
phi and ω scans	θ_max_ = 25.8°, θ_min_ = 1.9°
Absorption correction: multi-scan (*SADABS*; Bruker, 2000)	*h* = −21→21
*T*_min_ = 0.887, *T*_max_ = 0.941	*k* = −13→6
10338 measured reflections	*l* = −26→26

### Refinement

**Table:**

Refinement on *F*^2^	Primary atom site location: structure-invariant direct methods
Least-squares matrix: full	Secondary atom site location: difference Fourier map
*R*[*F*^2^ > 2σ(*F*^2^)] = 0.047	Hydrogen site location: inferred from neighbouring sites
*wR*(*F*^2^) = 0.110	H atoms treated by a mixture of independent and constrained refinement
*S* = 1.08	*w* = 1/[σ^2^(*F*_o_^2^) + (0.0527*P*)^2^] where *P* = (*F*_o_^2^ + 2*F*_c_^2^)/3
3867 reflections	(Δ/σ)_max_ = 0.022
300 parameters	Δρ_max_ = 0.80 e Å^−^^3^
13 restraints	Δρ_min_ = −0.48 e Å^−^^3^

### Special details

**Table:**

Geometry. All e.s.d.'s (except the e.s.d. in the dihedral angle between two l.s. planes) are estimated using the full covariance matrix. The cell e.s.d.'s are taken into account individually in the estimation of e.s.d.'s in distances, angles and torsion angles; correlations between e.s.d.'s in cell parameters are only used when they are defined by crystal symmetry. An approximate (isotropic) treatment of cell e.s.d.'s is used for estimating e.s.d.'s involving l.s. planes.
Refinement. Refinement of *F*^2^ against ALL reflections. The weighted *R*-factor *wR* and goodness of fit *S* are based on *F*^2^, conventional *R*-factors *R* are based on *F*, with *F* set to zero for negative *F*^2^. The threshold expression of *F*^2^ > σ(*F*^2^) is used only for calculating *R*-factors(gt) *etc*. and is not relevant to the choice of reflections for refinement. *R*-factors based on *F*^2^ are statistically about twice as large as those based on *F*, and *R*- factors based on ALL data will be even larger.

### Fractional atomic coordinates and isotropic or equivalent isotropic displacement parameters (Å^2^)

**Table:**

	*x*	*y*	*z*	*U*_iso_*/*U*_eq_	
Cd1	0.0000	0.74023 (3)	−0.2500	0.03203 (14)	
C1	0.1391 (2)	0.6471 (3)	−0.14433 (15)	0.0341 (8)	
C2	0.16577 (18)	0.6286 (3)	−0.07425 (14)	0.0304 (7)	
C3	0.21138 (19)	0.5274 (3)	−0.04962 (15)	0.0340 (8)	
H3A	0.2266	0.4705	−0.0762	0.041*	
C4	0.23363 (19)	0.5125 (3)	0.01481 (16)	0.0334 (8)	
C5	0.2124 (2)	0.5954 (3)	0.05570 (15)	0.0360 (8)	
H5	0.2276	0.5832	0.0990	0.043*	
C6	0.16780 (19)	0.6977 (3)	0.03069 (15)	0.0312 (7)	
C7	0.14483 (19)	0.7127 (3)	−0.03395 (15)	0.0316 (7)	
H7	0.1147	0.7807	−0.0505	0.038*	
C8	0.3530 (2)	0.3931 (3)	0.03346 (16)	0.0390 (8)	
C9	0.3967 (2)	0.2847 (3)	0.06802 (18)	0.0406 (9)	
C10	0.4431 (2)	0.2140 (4)	0.0395 (2)	0.0550 (11)	
H10	0.4453	0.2292	−0.0018	0.066*	
C11	0.4868 (3)	0.1191 (4)	0.0739 (2)	0.0638 (13)	
H11	0.5172	0.0701	0.0541	0.077*	
C12	0.4417 (3)	0.1640 (4)	0.1594 (2)	0.0609 (12)	
H12	0.4406	0.1472	0.2008	0.073*	
C13	0.3957 (3)	0.2595 (3)	0.1292 (2)	0.0532 (11)	
H13	0.3648	0.3054	0.1499	0.064*	
C14	0.1521 (2)	0.7883 (3)	0.13085 (16)	0.0372 (8)	
C15	0.1209 (2)	0.9006 (3)	0.15632 (15)	0.0352 (8)	
C16	0.1481 (3)	0.9300 (4)	0.21868 (17)	0.0524 (11)	
H16	0.1864	0.8819	0.2446	0.063*	
C17	0.1180 (3)	1.0315 (4)	0.24232 (18)	0.0534 (11)	
H17	0.1380	1.0517	0.2844	0.064*	
C18	0.0367 (2)	1.0727 (4)	0.14846 (17)	0.0487 (10)	
H18	−0.0018	1.1220	0.1236	0.058*	
C19	0.0636 (2)	0.9741 (3)	0.12058 (17)	0.0460 (9)	
H19	0.0433	0.9572	0.0782	0.055*	
N1	0.4871 (2)	0.0953 (3)	0.13331 (19)	0.0625 (10)	
N2	0.06210 (18)	1.1019 (3)	0.20837 (13)	0.0398 (7)	
N3	0.28104 (17)	0.4100 (3)	0.04134 (13)	0.0402 (7)	
H3	0.2624	0.3573	0.0632	0.048*	
N4	0.14702 (16)	0.7905 (3)	0.06830 (12)	0.0353 (7)	
H4	0.1288	0.8572	0.0491	0.042*	
O1	0.09323 (16)	0.7351 (2)	−0.16214 (11)	0.0429 (6)	
O2	0.16316 (16)	0.5753 (2)	−0.17948 (11)	0.0505 (7)	
O3	0.38414 (17)	0.4597 (2)	0.00154 (13)	0.0585 (8)	
O4	0.17829 (19)	0.7016 (2)	0.16454 (12)	0.0571 (8)	
O5	0.07154 (19)	0.6011 (3)	−0.29733 (14)	0.0501 (7)	
O6	0.1941 (3)	0.3124 (5)	0.1269 (2)	0.1053 (14)	
O7	0.2760 (3)	0.1472 (3)	0.20727 (17)	0.0782 (11)	
H5A	0.050 (2)	0.539 (3)	−0.3152 (17)	0.055 (13)*	
H7B	0.299 (3)	0.154 (6)	0.2454 (12)	0.12 (2)*	
H7A	0.283 (3)	0.073 (2)	0.198 (2)	0.11 (2)*	
H5B	0.109 (2)	0.575 (4)	−0.2698 (18)	0.088 (19)*	
H6B	0.150 (2)	0.325 (7)	0.135 (3)	0.16 (3)*	
H6A	0.207 (4)	0.243 (3)	0.143 (3)	0.112 (6)*	

### Atomic displacement parameters (Å^2^)

**Table:**

	*U*^11^	*U*^22^	*U*^33^	*U*^12^	*U*^13^	*U*^23^
Cd1	0.0418 (2)	0.0269 (2)	0.0257 (2)	0.000	0.00478 (15)	0.000
C1	0.0364 (19)	0.0347 (19)	0.0303 (18)	−0.0015 (16)	0.0064 (15)	−0.0028 (15)
C2	0.0296 (17)	0.0320 (17)	0.0274 (17)	0.0024 (14)	0.0027 (14)	0.0007 (13)
C3	0.038 (2)	0.0279 (18)	0.0353 (19)	0.0029 (15)	0.0069 (16)	−0.0035 (14)
C4	0.0333 (19)	0.0292 (18)	0.0377 (19)	0.0076 (14)	0.0082 (15)	0.0049 (14)
C5	0.043 (2)	0.0360 (19)	0.0276 (18)	0.0080 (16)	0.0061 (15)	0.0047 (14)
C6	0.0327 (18)	0.0293 (17)	0.0324 (18)	0.0043 (14)	0.0095 (15)	0.0024 (14)
C7	0.0304 (18)	0.0292 (17)	0.0348 (18)	0.0057 (14)	0.0069 (15)	0.0022 (14)
C8	0.045 (2)	0.0354 (19)	0.035 (2)	0.0108 (17)	0.0064 (17)	0.0046 (15)
C9	0.040 (2)	0.0330 (19)	0.047 (2)	0.0070 (16)	0.0064 (17)	0.0066 (16)
C10	0.055 (3)	0.049 (2)	0.062 (3)	0.016 (2)	0.015 (2)	0.010 (2)
C11	0.053 (3)	0.045 (2)	0.095 (4)	0.016 (2)	0.020 (3)	0.013 (2)
C12	0.054 (3)	0.063 (3)	0.060 (3)	0.001 (2)	0.003 (2)	0.024 (2)
C13	0.053 (2)	0.052 (3)	0.053 (3)	0.013 (2)	0.010 (2)	0.0152 (19)
C14	0.041 (2)	0.0374 (19)	0.0326 (19)	0.0035 (16)	0.0077 (16)	−0.0008 (15)
C15	0.043 (2)	0.0338 (18)	0.0314 (18)	0.0054 (16)	0.0139 (16)	0.0018 (14)
C16	0.066 (3)	0.049 (2)	0.038 (2)	0.023 (2)	0.0029 (19)	−0.0039 (18)
C17	0.071 (3)	0.054 (3)	0.030 (2)	0.014 (2)	0.0031 (19)	−0.0071 (18)
C18	0.056 (2)	0.053 (2)	0.037 (2)	0.023 (2)	0.0090 (18)	0.0032 (18)
C19	0.054 (2)	0.053 (2)	0.0300 (19)	0.014 (2)	0.0076 (17)	−0.0028 (17)
N1	0.044 (2)	0.050 (2)	0.090 (3)	0.0065 (17)	0.006 (2)	0.027 (2)
N2	0.0495 (19)	0.0383 (17)	0.0319 (16)	0.0079 (14)	0.0104 (14)	0.0013 (13)
N3	0.0494 (19)	0.0319 (16)	0.0403 (18)	0.0120 (14)	0.0127 (15)	0.0111 (12)
N4	0.0453 (18)	0.0305 (15)	0.0307 (16)	0.0105 (13)	0.0104 (14)	0.0038 (12)
O1	0.0536 (16)	0.0396 (14)	0.0300 (13)	0.0159 (12)	−0.0009 (11)	−0.0014 (10)
O2	0.0682 (19)	0.0500 (16)	0.0320 (14)	0.0208 (14)	0.0094 (13)	−0.0039 (12)
O3	0.0618 (18)	0.0565 (18)	0.0640 (18)	0.0204 (15)	0.0286 (15)	0.0286 (15)
O4	0.094 (2)	0.0429 (15)	0.0350 (15)	0.0257 (16)	0.0179 (15)	0.0076 (12)
O5	0.0571 (19)	0.0382 (16)	0.0509 (18)	0.0037 (13)	0.0048 (14)	−0.0158 (13)
O6	0.105 (3)	0.114 (3)	0.112 (3)	0.032 (3)	0.057 (3)	0.061 (3)
O7	0.124 (3)	0.058 (2)	0.053 (2)	0.027 (2)	0.021 (2)	−0.0028 (17)

### Geometric parameters (Å, °)

**Table:**

Cd1—O1	2.210 (2)	C11—H11	0.9300
Cd1—O1^i^	2.210 (2)	C12—N1	1.318 (5)
Cd1—N2^ii^	2.331 (3)	C12—C13	1.382 (5)
Cd1—N2^iii^	2.331 (3)	C12—H12	0.9300
Cd1—O5^i^	2.358 (3)	C13—H13	0.9300
Cd1—O5	2.358 (3)	C14—O4	1.217 (4)
C1—O2	1.239 (4)	C14—N4	1.351 (4)
C1—O1	1.252 (4)	C14—C15	1.498 (5)
C1—C2	1.507 (4)	C15—C16	1.372 (5)
C2—C7	1.379 (4)	C15—C19	1.374 (5)
C2—C3	1.391 (4)	C16—C17	1.376 (5)
C3—C4	1.380 (4)	C16—H16	0.9300
C3—H3A	0.9300	C17—N2	1.325 (5)
C4—C5	1.382 (4)	C17—H17	0.9300
C4—N3	1.428 (4)	C18—N2	1.319 (4)
C5—C6	1.394 (4)	C18—C19	1.371 (5)
C5—H5	0.9300	C18—H18	0.9300
C6—C7	1.386 (4)	C19—H19	0.9300
C6—N4	1.404 (4)	N2—Cd1^ii^	2.331 (3)
C7—H7	0.9300	N3—H3	0.8600
C8—O3	1.222 (4)	N4—H4	0.8600
C8—N3	1.330 (4)	O5—H5A	0.829 (19)
C8—C9	1.507 (5)	O5—H5B	0.83 (3)
C9—C13	1.372 (6)	O6—H6B	0.84 (2)
C9—C10	1.373 (6)	O6—H6A	0.83 (2)
C10—C11	1.394 (5)	O7—H7B	0.84 (2)
C10—H10	0.9300	O7—H7A	0.85 (2)
C11—N1	1.325 (5)		
			
O1—Cd1—O1^i^	177.13 (12)	C11—C10—H10	120.7
O1—Cd1—N2^ii^	89.86 (9)	N1—C11—C10	123.1 (4)
O1^i^—Cd1—N2^ii^	92.25 (10)	N1—C11—H11	118.5
O1—Cd1—N2^iii^	92.25 (10)	C10—C11—H11	118.5
O1^i^—Cd1—N2^iii^	89.86 (9)	N1—C12—C13	124.4 (4)
N2^ii^—Cd1—N2^iii^	85.36 (15)	N1—C12—H12	117.8
O1—Cd1—O5^i^	87.95 (10)	C13—C12—H12	117.8
O1^i^—Cd1—O5^i^	90.21 (10)	C9—C13—C12	118.2 (4)
N2^ii^—Cd1—O5^i^	87.17 (11)	C9—C13—H13	120.9
N2^iii^—Cd1—O5^i^	172.53 (10)	C12—C13—H13	120.9
O1—Cd1—O5	90.21 (10)	O4—C14—N4	123.5 (3)
O1^i^—Cd1—O5	87.95 (10)	O4—C14—C15	121.6 (3)
N2^ii^—Cd1—O5	172.53 (10)	N4—C14—C15	114.9 (3)
N2^iii^—Cd1—O5	87.17 (11)	C16—C15—C19	117.8 (3)
O5^i^—Cd1—O5	100.30 (15)	C16—C15—C14	119.2 (3)
O2—C1—O1	125.3 (3)	C19—C15—C14	123.0 (3)
O2—C1—C2	118.7 (3)	C15—C16—C17	119.1 (4)
O1—C1—C2	116.1 (3)	C15—C16—H16	120.5
C7—C2—C3	119.5 (3)	C17—C16—H16	120.5
C7—C2—C1	119.8 (3)	N2—C17—C16	123.5 (4)
C3—C2—C1	120.7 (3)	N2—C17—H17	118.2
C4—C3—C2	119.0 (3)	C16—C17—H17	118.2
C4—C3—H3A	120.5	N2—C18—C19	124.0 (3)
C2—C3—H3A	120.5	N2—C18—H18	118.0
C3—C4—C5	122.1 (3)	C19—C18—H18	118.0
C3—C4—N3	120.2 (3)	C15—C19—C18	118.9 (3)
C5—C4—N3	117.7 (3)	C15—C19—H19	120.5
C4—C5—C6	118.5 (3)	C18—C19—H19	120.5
C4—C5—H5	120.7	C12—N1—C11	117.0 (4)
C6—C5—H5	120.7	C18—N2—C17	116.7 (3)
C7—C6—C5	119.6 (3)	C18—N2—Cd1^ii^	119.1 (2)
C7—C6—N4	117.5 (3)	C17—N2—Cd1^ii^	124.0 (2)
C5—C6—N4	122.9 (3)	C8—N3—C4	122.4 (3)
C2—C7—C6	121.2 (3)	C8—N3—H3	118.8
C2—C7—H7	119.4	C4—N3—H3	118.8
C6—C7—H7	119.4	C14—N4—C6	128.2 (3)
O3—C8—N3	124.2 (3)	C14—N4—H4	115.9
O3—C8—C9	120.3 (3)	C6—N4—H4	115.9
N3—C8—C9	115.5 (3)	C1—O1—Cd1	125.4 (2)
C13—C9—C10	118.7 (3)	Cd1—O5—H5A	120 (3)
C13—C9—C8	121.5 (3)	Cd1—O5—H5B	109 (3)
C10—C9—C8	119.7 (4)	H5A—O5—H5B	105 (4)
C9—C10—C11	118.6 (4)	H6B—O6—H6A	104 (6)
C9—C10—H10	120.7	H7B—O7—H7A	104 (5)
			
O2—C1—C2—C7	175.0 (3)	O4—C14—C15—C19	−151.8 (4)
O1—C1—C2—C7	−5.5 (5)	N4—C14—C15—C19	26.8 (5)
O2—C1—C2—C3	−5.1 (5)	C19—C15—C16—C17	−0.9 (6)
O1—C1—C2—C3	174.5 (3)	C14—C15—C16—C17	−178.5 (4)
C7—C2—C3—C4	1.0 (5)	C15—C16—C17—N2	1.8 (7)
C1—C2—C3—C4	−179.0 (3)	C16—C15—C19—C18	0.3 (6)
C2—C3—C4—C5	−0.4 (5)	C14—C15—C19—C18	177.8 (4)
C2—C3—C4—N3	−179.4 (3)	N2—C18—C19—C15	−0.5 (6)
C3—C4—C5—C6	−0.7 (5)	C13—C12—N1—C11	−1.2 (7)
N3—C4—C5—C6	178.4 (3)	C10—C11—N1—C12	1.9 (7)
C4—C5—C6—C7	1.1 (5)	C19—C18—N2—C17	1.3 (6)
C4—C5—C6—N4	−176.4 (3)	C19—C18—N2—Cd1^ii^	−173.3 (3)
C3—C2—C7—C6	−0.6 (5)	C16—C17—N2—C18	−1.9 (6)
C1—C2—C7—C6	179.4 (3)	C16—C17—N2—Cd1^ii^	172.4 (3)
C5—C6—C7—C2	−0.5 (5)	O3—C8—N3—C4	−2.7 (6)
N4—C6—C7—C2	177.1 (3)	C9—C8—N3—C4	176.3 (3)
O3—C8—C9—C13	135.5 (4)	C3—C4—N3—C8	62.3 (5)
N3—C8—C9—C13	−43.5 (5)	C5—C4—N3—C8	−116.8 (4)
O3—C8—C9—C10	−40.0 (5)	O4—C14—N4—C6	1.7 (6)
N3—C8—C9—C10	141.0 (4)	C15—C14—N4—C6	−176.9 (3)
C13—C9—C10—C11	0.4 (6)	C7—C6—N4—C14	169.1 (3)
C8—C9—C10—C11	176.0 (4)	C5—C6—N4—C14	−13.4 (6)
C9—C10—C11—N1	−1.5 (7)	O2—C1—O1—Cd1	31.5 (5)
C10—C9—C13—C12	0.2 (6)	C2—C1—O1—Cd1	−148.0 (2)
C8—C9—C13—C12	−175.3 (4)	N2^ii^—Cd1—O1—C1	160.5 (3)
N1—C12—C13—C9	0.2 (7)	N2^iii^—Cd1—O1—C1	−114.2 (3)
O4—C14—C15—C16	25.7 (6)	O5^i^—Cd1—O1—C1	73.3 (3)
N4—C14—C15—C16	−155.7 (3)	O5—Cd1—O1—C1	−27.0 (3)

Symmetry codes: (i) −*x*, *y*, −*z*−1/2; (ii) −*x*, −*y*+2, −*z*; (iii) *x*, −*y*+2, *z*−1/2.

### Hydrogen-bond geometry (Å, °)

**Table:**

*D*—H···*A*	*D*—H	H···*A*	*D*···*A*	*D*—H···*A*
N3—H3···O6	0.86	2.10	2.885 (5)	151
N4—H4···O3^iv^	0.86	2.26	3.096 (4)	164
O5—H5B···O2	0.83 (3)	1.98 (3)	2.710 (4)	145 (4)
O5—H5A···N1^v^	0.83 (2)	2.00 (2)	2.825 (4)	172 (4)
O6—H6B···O5^vi^	0.84 (2)	2.40 (4)	3.163 (6)	151 (6)
O6—H6A···O7	0.82 (3)	1.92 (4)	2.681 (6)	148 (5)
O7—H7A···O2^vii^	0.85 (2)	1.96 (3)	2.767 (4)	162 (5)
O7—H7B···O4^viii^	0.84 (2)	1.99 (3)	2.791 (4)	159 (6)

Symmetry codes: (iv) −*x*+1/2, −*y*+3/2, −*z*; (v) *x*−1/2, −*y*+1/2, *z*−1/2; (vi) *x*, −*y*+1, *z*+1/2; (vii) −*x*+1/2, −*y*+1/2, −*z*; (viii) −*x*+1/2, *y*−1/2, −*z*+1/2.

## References

[bb1] Bent, H. A. (1968). *Chem. Rev.* **68**, 587–648.

[bb2] Biradha, K., Sarkar, M. & Rajput, L. (2006). *Chem. Commun.* pp. 4169–4179. 10.1039/b606184b 17031423

[bb3] Bruker (2000). *SMART*, *SAINT *and *SADABS* Bruker AXS Inc., Madison, Wisconsin, USA.

[bb4] Chen, M. S., Chen, S. S., Okamuraz, T.-A., Su, Z., Sun, W. Y. & Ueyama, N. (2009). *J. Coord. Chem.* **62**, 2421–2428.

[bb5] Cheng, D. P., Khan, M. A. & Houser, R. P. (2002). *J. Chem. Soc. Dalton Trans.* pp. 4555–4560.

[bb6] Eddaoudi, M., Moler, D. B., Li, H. L., Chen, B. L., Reineke, T. M., O’Keeffe, M. & Yaghi, O. M. (2001). *Acc. Chem. Res.* **34**, 319–330. 10.1021/ar000034b 11308306

[bb7] Evans, O. R. & Lin, W. (2002). *Acc. Chem. Res.* **35**, 511–522. 10.1021/ar0001012 12118990

[bb8] Go, Y. B., Wang, X. Q., Anokhina, E. V. & Jacobson, A. J. (2004). *Inorg. Chem.* **43**, 5360–5367. 10.1021/ic049341u 15310214

[bb9] Huyskens, P. L. (1977). *J. Am. Chem. Soc.* **99**, 2578–2582.

[bb10] Kitagawa, S., Kitaura, R. & Noro, S. (2004). *Angew. Chem. Int. Ed.* **43**, 2334–2375. 10.1002/anie.200300610 15114565

[bb11] Lee, C. M. & Kumler, W. D. (1962). *J. Am. Chem. Soc.* **84**, 571–578.

[bb12] Moulton, B. & Zaworotko, M. J. (2001). *Chem. Rev.* **101**, 1629–1658. 10.1021/cr9900432 11709994

[bb13] Sheldrick, G. M. (2008). *Acta Cryst.* A**64**, 112–122. 10.1107/S0108767307043930 18156677

[bb14] Wang, Y., Huang, Y. Q., Liu, G. X., Okamuraz, T.-A., Doi, M., Sheng, Y. W., Sun, W. Y. & Ueyama, N. (2007). *Chem. Eur. J.* **13**, 7523–7531. 10.1002/chem.200700133 17582817

[bb15] Wu, S. T., Ma, L. Q., Long, L. S., Zheng, L. S. & Lin, W. B. (2009). *Inorg. Chem.* **48**, 2436–2442. 10.1021/ic801631w 19226169

[bb16] Yaghi, O. M., O’Keeffe, M., Ockwig, N. W., Chae, H. K., Eddaoudi, M. & Kim, J. (2003). *Nature (London)*, **423**, 705–714. 10.1038/nature01650 12802325

[bb17] Zhang, L. Y., Liu, G. F., Zheng, S. L., Ye, B. H. & Chen, X. M. (2003). *Eur. J. Inorg. Chem.* pp. 2965–2971.

